# Extracellular Hsp90 and TGFβ regulate adhesion, migration and anchorage independent growth in a paired colon cancer cell line model

**DOI:** 10.1186/s12885-017-3190-z

**Published:** 2017-03-16

**Authors:** Jo-Anne de la Mare, Tamarin Jurgens, Adrienne L. Edkins

**Affiliations:** The Biomedical Biotechnology Research Unit, Department of Biochemistry and Microbiology, Rhodes University, Grahamstown, 6139 South Africa

**Keywords:** TGF-β pathway, Hsp90, Colon cancer, Migration, Anchorage-independent growth

## Abstract

**Background:**

Tumour metastasis remains the major cause of death in cancer patients and, to date, the mechanism and signalling pathways governing this process are not completely understood. The TGF-β pathway is the most commonly mutated pathway in cancer, however its role in cancer progression is controversial as it can function as both a promoter and a suppressor of metastasis. Although previous studies have suggested a role for the molecular chaperone Hsp90 in regulating the TGF-β pathway, the level at which this occurs as well as the consequences in terms of colon cancer metastasis are unknown.

**Methods:**

The paired SW480 and SW620 colon cancer cell lines, derived from a primary tumour and its lymph node metastasis, respectively, were used as an in vitro model to study key cellular processes required for metastasis. The status of the TGF-β pathway was examined in these cells using ELISA, flow cytometry, western blot analysis and confocal microscopy. Furthermore, the effect of addition or inhibition of the TGF-β pathway and Hsp90 on adhesion, migration and anchorage-independent growth, was determined in the cell lines.

**Results:**

When comparing the canonical TGF-β1 pathway in the genetically paired cell lines our data suggests that this pathway may be constitutively active in the SW620 metastasis-derived cell line and not the SW480 primary tumour-derived line. In addition, we report that, when present in combination, TGF-β1 and Hsp90β stimulate anchorage-independent growth, reduce adhesion and stimulate migration. This effect is potentiated by inhibition of the TGF-β1 receptor and occurs via an alternate TGF-β1 pathway, mediated by αvβ6 integrin. Interestingly, in the SW620 cells, activation of this alternate TGF-β1 signalling machinery does not appear to require inhibition of the canonical TGF-β1 receptor, which would allow them to respond more effectively to the pro-metastasis stimulus of a combination of Hsp90β and TGF-β1 and this could account for the increased migratory capacity of these cells.

**Conclusions:**

In this study we report an apparent synergy between TGF-β1 and Hsp90β in stimulating migratory behaviour of colon cancer cells when signalling occurs via αvβ6 integrin as opposed to the canonical TGF-β1 pathway.

**Electronic supplementary material:**

The online version of this article (doi:10.1186/s12885-017-3190-z) contains supplementary material, which is available to authorized users.

## Background

According to the latest available statistics, colorectal cancer is the second highest cause of cancer related deaths in the USA [1]. A potential reason for this high fatality rate is the fact that this form of the disease is highly metastatic [2, 3]. Despite the fact that the mechanisms underlying tumour metastasis have been a major focus in cancer research in recent years, the spread of cancers to secondary sites in the body remains the leading cause of mortality [3, 4].

The “seeds and soil” theory of metastasis proposed by Paget over a century ago, suggests that metastasis is dependent on characteristics of both the migrating tumour cells (seeds) and the local environment (soil) [5]. The microenvironment refers to the complex milieu surrounding tumour cells and is composed of a unique combination of non-cancerous cells including fibroblasts, endothelial and various immune cells as well as chemical messengers in the form of cytokines and chemokines [6]. There is a growing appreciation of the role of the microenvironment, the “soil” in Paget’s theory of metastasis, in the development and spread of cancers as evidenced by the exponential increase in the number of research articles on this topic in recent years [7].

Of particular interest to this study, the microenvironmental niche element transforming growth factor-β (TGF-β) has been found to represent the most commonly altered signalling pathway in cancer [8–10]. The TGF-β superfamily consists of a number of related ligands, namely TGF-β1, TGF-β2, TGF-β3, bone morphogenetic proteins (BMP), activin, nodal and growth and differentiation factors (GDFs) each with specific receptor affinities. In the canonical TGF-β pathway, the TGF-β1 ligand binds to the type II receptor (TGF-βRII) which then recruits the type I receptor (TGF-βRI) forming a heterodimeric complex which stimulates receptor-associated protein kinase activity [11, 12]. This phosphorylates the transcription factors Smad2 and Smad3, resulting in the binding of Smad2 and Smad3 to Smad4. This complex translocates into the nucleus where it regulates the expression of a large cohort of genes responsible for controlling key cellular processes such as proliferation, differentiation and survival [12]. Although TGF-β has been implicated in colon cancer metastasis [13], the existing data is contradictory and controversial, with some groups claiming that the protein inhibits metastasis [14], while others report that it promotes metastasis [15].

Tumour cells, particularly migrating cells, are exposed to a variety of physiological stresses and produce an accumulation of overexpressed and mutated proteins requiring an upregulation of molecular chaperones in order to overcome these stresses [16]. One such chaperone is heat shock protein 90 (Hsp90) which has also been referred to as the cancer chaperone due to its large cohort of oncogenic client proteins and 2-10 fold upregulation in cancer cells [17]. Hsp90 is a drug target with a number of inhibitors targeting this protein in clinical trials [18, 19]. The chaperone has two cytosolic isoforms; Hsp90α, which is inducible yet nonessential in the mouse and Hsp90β, the constitutive and essential isoform of the protein [20, 21]. The vast majority of studies regarding the role of Hsp90 in cancer biology have been carried out in the intracellular compartments of the cell [22]. In the last decade, however, Hsp90 has increasingly been found to activate signalling pathways outside the cell, on the cell surface facing the extracellular space and in the extracellular media [21, 23–30]. Hsp90β has been shown to negatively regulate TGF-β activation in the extracellular space of osteosarcoma cells in vitro by binding to the latency-associated peptide (LAP) of inactive TGF-β, while itself being upregulated by active TGF-β, resulting in a negative feedback loop [29]. In addition, it has been reported that Hsp90 regulates TGF-β signalling by binding directly to both receptors, TGF-βRI and TGF-βRII, preventing their degradation in non-malignant human cells [31].

Extracellular TGF-β and Hsp90 both stimulate migration of cancer cells [30, 32–35], however a synergy between these two proteins in modulating in vitro processes linked to metastasis has not been demonstrated despite the fact that they bind common receptors [31].

## Methods

### Reagents

Leibovitz’s L-15 medium with GlutaMAX™, 10 x Trypsin-Ethylenediaminetetraacetic acid (EDTA), Penicillin/Streptomycin/Amphotericin (PSA) solution and Hoescht-33342 dye were from Gibco, Invitrogen. Heat-inactivated fetal bovine serum (FBS) was from Celtic Molecular Diagnostics and Epidermal growth factor (EGF), basic fibroblast growth factor (bFGF) and Accutase® were purchased from Sigma-Aldrich. Insulin was obtained from NovoRapid (Novo Nordisk Pharmaceuticals). Hybond nitrocellulose membrane and Clarity Western Enhanced Chemiluminescence [ECL] substrate was from Bio-Rad (UK). Protein A/G PLUS-Agarose (sc-2003) was from Santa Cruz Biotechnology (USA). SB431542 (1614) was from Tocris Bioscience, while 3,3’, 5,5’ Tetramethyl-benzidine (TMB) substrate, dimethyl sulfoxide (DMSO), novobiocin (N1838), 5-fluorouracil (5-FU) and oxaliplatin were from Sigma-Aldrich. Recombinant native endotoxin-free human Hsp90β protein (SPR-102C) was from StressMarq Biosciences Inc., while recombinant endotoxin-free human TGF-β1 (carrier-free) (580704) was from BioLegend and bovine serum albumin (BSA) (10735078001) was from Roche. Mouse anti-human Hsp90α/β [F-8] (sc-13119), goat anti-human Hsp90α/β [N17] (sc-1055), HRP-conjugated donkey anti-mouse (sc-2314), mouse anti-human TGF-β1 [500-M66] (sc-65378), goat anti-human p-Smad2/3 (Ser 423/425) (sc-11769) and rabbit anti-human Smad2/3 (sc-8332) antibodies were from Santa Cruz. The Alexa-Fluor-550 conjugated donkey anti-rat (A21208) antibody was from Invitrogen, while the HRP-conjugated mouse anti-alpha tubulin (ab40742) and Alexa-Fluor-550 conjugated donkey anti-mouse (ab96876), donkey anti-rabbit IgG DyLight 550 (DY550) (ab96892), donkey anti-goat IgG DY660 (ab96934) and anti-human αvβ6 integrin (ab77906) antibodies were from Abcam. The fluorescein isothiocyanate (FITC)-conjugated mouse anti-human TGF-βRII (FAB241F) antibody was from R&D systems and the rat anti-human Hsp90α antibody (ADI-SPA-840-F) was from Enzo Life Sciences. The mouse anti-human Hsp90β (SMC-107A) antibody was from StressMarq Biosciences Inc. and the rabbit anti-histone H3 (sc-10809) antibody was from Cell Signaling Technologies. The FITC-conjugated mouse IgG1 isotype control was from BD Biosciences. The MTT Cell Proliferation kit 1 was from Roche.

### Cell lines

The paired colon cancer cell lines SW480 (ECACC: 87092801, colon adenocarcinoma) and SW620 (ECACC: 87051203, lymph node metastasis) were purchased from the European Collection of Cell Cultures (ECACC) and maintained in L-15 Medium with GlutaMAX™ supplemented with 10% [v/v] fetal calf serum (Lonza) and 100 U/ml Penicillin, 100 μg/ml Streptomycin and 12.5 μg/ml Amphotericin at 37 °C in a humidified incubator without the introduction of CO_2_.

### Sodium Dodecyl Sulphate-Polyacrylamide Gel Electrophoresis (SDS PAGE) and Western blot analysis

Unless stated otherwise, whole cell lysates were prepared from confluent 6-well plates (Nunc) by scraping into lysis buffer (0.05 M Tris-HCl, 10% [v/v] glycerol, 2% [w/v] SDS). The total protein content of cell lysates was determined using a NanoDrop 2000™ spectrophotometer (Thermo Scientific). The method described by Laemmli (1970) [36] was used to separate proteins by denaturing electrophoresis according to size. Western blotting was performed according to Towbin et al. (1979) [37]. Proteins were visualised using Clarity Western Enhanced Chemiluminescence [ECL] substrate and detected using the ChemiDoc™ XRS+ system (Bio-Rad) or the film exposure method (Agfa HealthCare). Where relevant, lysates were probed for histone H3 (rabbit ant-histone H3) or tubulin (mouse anti-alpha tubulin) as a loading control.

### Confocal microscopy

Gelatin (5% [w/v]) was used to coat 15-well μ-Slide angiogenesis plates (81506, Ibidi). SW480 and SW620 cells were seeded onto μ-Slide plates and fixed with methanol and incubated with appropriate primary antibodies followed by the respective secondary antibodies as described in figure legends. Nuclei were stained with 1 μg/ml Hoechst-33342 dye, after which Dako mounting medium was added to each well. Immunofluorescence was detected using the Zeiss LSM 780 laser scanning confocal microscope and the images analysed using Zen Lite Software 2012 (Zeiss). Images were captured using the 63x oil objective. Colocalisation between the nucleus and pSMAD2/3 staining was performed using the Image J colocalisation plug-in (National Institutes of Health). The proportion of total pSMAD2/3 that is found in the nucleus was determined by developing profiles of the intensity of pSMAD2/3 in a line through the cells using Zen Lite Software.

### Enzyme-linked Immunosorbent Assays (ELISAs)

Cells were seeded into 6-well plates and allowed to adhere overnight. The spent media was removed and centrifuged to remove cell debris. A human TGF-β1 DuoSet Development ELISA kit (R&D Systems Inc.) was utilised to analyse the expression of TGF-β1 in the cell culture supernatant (spent media) of the SW480 and SW620 cell lines. This was carried out according to manufacturer’s specifications. Samples of spent medium were assayed in triplicate and the concentration of TGF-β1 determined from a standard curve generated by assaying purified recombinant human TGF-β1 diluted to an eight-point range between 0 pg/ml and 2000 pg/ml in duplicate. Hsp90β secretion by cells was determined by ELISA from spent media according to Hunter et al., (2014) [26]. Briefly, spent media or pure protein in buffer A (20 mM Tris-HCl, 150 mM NaCl, pH 7.4, 1 mM ATP, 5 mM CaCl2, 0.05% [v/v] Tween 20) was incubated in a high binding 96-well microplate (655061, Greiner Bio-One, UK) overnight. Wells were blocked with 3% (w/v) bovine serum albumin (BSA) in buffer A for 1 h and washed with 1% (w/v) BSA in buffer A, before incubating with mouse anti-Hsp90β in buffer A (1:1000) for 2 h. After further washing, incubation with rabbit anti-mouse IgG-HRP secondary antibody in buffer A (1:10 000) was carried out for 1 h. Detection was carried out using TMB substrate (in 0.05 M Phosphate-Citrate Buffer [0.2 M Na_2_HPO_4_, 0.1 M citric acid, pH 5.0] with 2 μl of 30% [v/v] hydrogen peroxide) and quantification was carried out from a standard curve using recombinant Hsp90β at a six point concentration range from 0 to 500 ng/ml.

### Surface staining of cells for flow cytometry

Cells were harvested using Accutase® solution (Sigma-Aldrich), collected by centrifugation and resuspended to a final concentration of 5 × 10^6^ cells/ml. Thereafter 1 μg fluorescein isothiocyanate (FITC)-conjugated mouse anti-human TGF-βRII antibody was added to 1 × 10^6^ cells and incubated at 4 °C for 1 h in the dark. The stained cell suspension was washed, collected by centrifugation and resuspended in phosphate-buffered saline. The stained cells were analysed using the 488 nm laser of a FACSAria™ III flow cytometer (BD Biosciences) using wavelengths of 488 nm and 519 nm for excitation and emission, respectively. A FITC-conjugated mouse IgG1 isotype control was used to account for any non-specific binding of primary antibodies. A further negative control without primary antibody was used to assess cell autofluorescence. Data was analysed using FlowJo software version 10.0.4 (Tree Star Inc., 2013).

### Crystal violet adhesion assay

SW480 and SW620 cells were seeded at a density of 1.2 x 10^5^ cells/well into a 96-well plate and treated with combinations of TGF-β1, Hsp90β, SB431542, novobiocin and αvβ6 integrin blocking antibody as indicated in figure legends. The cells were left to adhere for 8 h at 37 °C before removing the media and washing the wells with PBS three times. Adherent cells were fixed with 4% [v/v] paraformaldehyde in PBS before washing with deionised water and staining with 10% [w/v] crystal violet in 5% [v/v] ethanol. Wells were again washed with deionised water, allowed to air-dry and crystal violet dye solubilised in 5% [w/v] SDS and 1% [v/v] Triton-X100. Absorbance was read at 590 nm using a microtitre plate reader (PowerWaveXTM, BioTek).

### Migration assay

SW480 and SW620 cells were seeded at a density of 1.2 x 10^4^ cells into each individual well of a micro-insert 4-well chamber (80409, Ibidi) and left to adhere overnight. Each insert was filled with L-15 medium containing combinations of TGF-β1, Hsp90β, SB431542, novobiocin or an αvβ6 integrin blocking antibody as indicated in figure legends. After 24 h, the inserts were lifted and the cell area was washed four times to remove floating cells. Photos of the intersection between the vertical and horizontal wounds generated by the micro-inserts were taken under a 10x objective using an iPhone 5 in conjunction with the SkyLight microscope adapter at 0, 12, and 24 h. Images were analysed in Image J (National Institutes of Health (NIH)) to determine the number of cells in the wound area (calculated as particles per mm^2^).

### Anchorage-independent tumoursphere assay

The ability of colon cancer cell lines to grow as tumourspheres in suspension was determined as previously described [38]. Briefly, cells were lifted with trypsin, washed with phosphate-buffered saline (PBS) and passed through a 40 μM cell strainer (BD Biosciences) to produce a single cell suspension. Cells were resuspended in anchorage-independent growth (AIG) medium containing DMEM with Glutamax™ supplemented with 1% (v/v) PSA, 2% (v/v) B-27 supplement, 20 ng/ml EGF and bFGF, 4 ng/ml heparin and 10 μg/ml insulin and seeded into ultralow attachment plates (Corning). Seeding was carried out at a density of 100 cells per well in a 96-well plate for analysis of effects of proteins/compounds on tumoursphere formation and 2000 cells/well in a 96-well plate for collection of spent media. Tumourspheres were fed every 3 days by the addition of 50 μl of fresh AIG medium to each well and processed on Day 7 post seeding.

### Chemosensitivity studies

SW480 and SW620 cells were seeded into anchorage-independent conditions as described above and treated with either 2 ng/ml TGF-β1, 20 ng/ml Hsp90β, 100 nM SB431542 or 100 μM novobiocin. After one week, tumourspheres were dissociated with Accutase® (Sigma-Aldrich) solution and reseeded into adherent condition in a 96 well plate at a density of 1.2 x 10^5^ cells/ml, allowed to adhere overnight and then treated with either 75 μM 5-FU or 550 μM oxaliplatin for 72 h. Cell viability was assessed compared to an untreated control for each pre-treatment using a MTT Cell Proliferation kit (Roche) according to manufacturer’s specifications as previously described [39].

### Statistical analysis

Statistical analysis was carried out using GraphPad Prism 4.03 software (GraphPad Inc., 2005). A Students *t*-test was performed when comparing single data sets between the two cell lines, while two-way ANOVA with Bonferroni post-tests were performed where data for a number of treatments were being compared.

## Results

### Alterations in the TGF-β pathway in a genetically paired cell line model

**Fig. 1:**
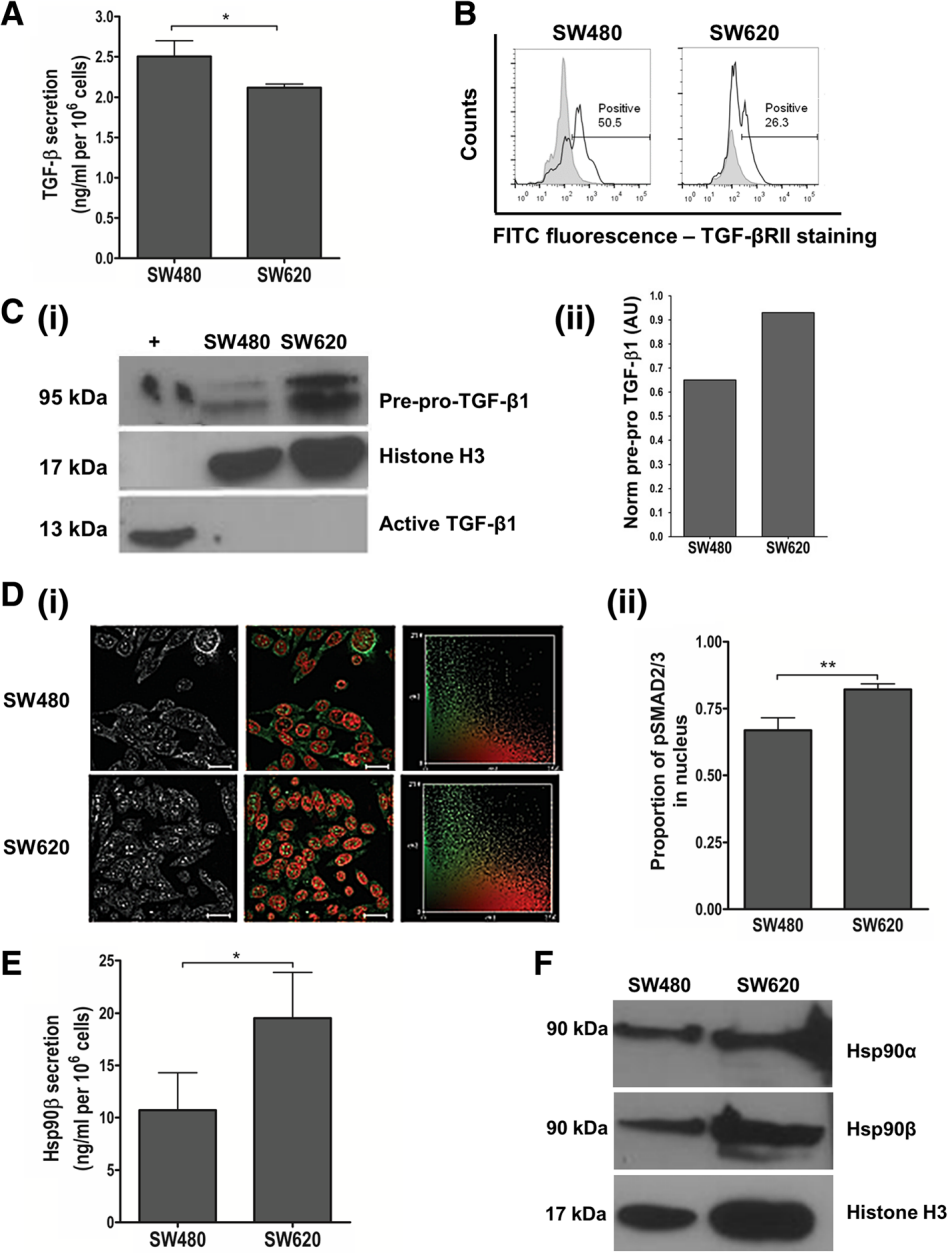
Investigating the status of the canonical TGF-β pathway and levels of Hsp90 in the genetically paired SW480 primary and SW620 secondary tumour-derived colon cancer cell lines. **a** The secretion of TGF-β1 by SW480 and SW620 cells was determined by means of an ELISA to quantify the levels of extracellular TGF-β1 in spent culture medium for a given cell number after 12 h incubation. Data represent the average of three independent experiments performed in triplicate and error bars indicate the standard error in the mean. **b** The levels of TGF-βRII receptor on the cell surface was determined by flow cytometry using a fluorescein isothiocyanate-conjugated antibody. Data was analysed using FlowJo software and gating carried out according to the IgG1 isotype control. Histograms show a shift in fluorescence for each sample (black line, unshaded) compared to the isotype control (grey shading) and the percentage of positive events is indicated for each sample. Data are representative of three independent experiments showing consistent results. **c** Intracellular levels of TGF-β1 in SW480 and SW620 cells were determined by (i) western blot analysis and compared to a histone loading control. The images are cropped from the full length western blot provided as Additional file 1. (ii) The normalized levels of pre-pro TGFβ1 from the western blot in (i) were determined by densitometry using ImageJ and calculated according the histone loading control. AU: arbitrary units. The positive control represents commercially obtained recombinant human TGFβ1 in its active form. **d** Activation of the TGF-β canonical pathway was determined by the phosphorylation and nuclear localization of Smad2/3. SW480 and SW620 cells were stained for pSMAD2/3 and nuclei were stained with Hoescht-33342. Immunofluorescence was detected using the Zeiss LSM 510 Meta laser scanning confocal microscope and the images analysed using Axiovision LE 4.7.1 software (Zeiss). (i) The first column shows the level of pSmad2/3 staining in the cells, pseudocoloured to white, while the second column shows a merged image of the nucleus pseudo-coloured to red and the pSMAD2/3 staining pseudocoloured to green. The third column shows frequency scattergrams obtained using colocalisation analysis on Image J,. Scale bars represent 20 μm. (ii) Graphs generated from profiles of the nucleus and pSMAD2/3 within individual cells analysed using Zen Lite Software showing the proportion of total pSMAD2/3 that is found in the nucleus. In all cases, analyses were performed on triplicate images containing at least 10 cells per image **e** The secretion of Hsp90β by SW480 and SW620 cells was quantified by means of an ELISA on spent culture medium after 12 h incubation. The data are representative of two individual experiments performed in triplicate and showing consistent results. **f** Whole cell lysates from SW480 and SW620 cells were analysed for Hsp90α and Hsp90β expression using western blot analysis. Where relevant, data was analysed using GraphPad Prism 4.03 software with errors bars indicating the standard error in the mean and a students *t*-test was performed to assess statistical significance. (*p < 0.05, **p < 0.01)

We analysed the status of the TGF-β pathway in the SW480 cell line derived from a primary colon adenocarcinoma and its SW620 lymph node metastasis-derived counterpart (Fig. 1). Analysis of the spent media of the cell lines by ELISA revealed that SW480 primary tumour cells and their SW620 secondary tumour counterparts secrete similar levels of TGF-β1 (2.50 ± 0.12 vs. 2.12 ± 0.05 ng/ml, Fig. 1a). Analysis of the cell surface receptors by flow cytometry showed that SW480 cells display twice the level of TGF-βRII compared to SW620 cells (Fig. 1b). SW620 cells, on the other hand, display greater levels of intracellular TGF-β1, specifically in the form of pre-pro-TGF-β1, compared to SW480 cells (Fig. 1c. Images cropped from the original full length western blots provided in Additional file 1). Confocal analysis revealed that SW620 cells also have a greater proportion of nuclear pSmad2/3 than SW480 cells (Fig. 1d), suggesting greater pathway activation in the metastatic cell line. Due to the fact that extracellular Hsp90 has been linked to the TGF-β pathway and is able to bind both TGF-βRI and TGF-βRII [31], we also examined the extracellular levels of Hsp90 in the spent medium of SW480 and SW620 cells. While we were unable to detect Hsp90α in the spent media (data not shown), we found that SW620 cells secrete significantly more Hsp90β than SW480 cells (19.51 ± 4.35 vs. 10.70 ± 3.60 ng/ml, respectively; Fig. 1e). In addition, we noted that the levels of Hsp90β secreted by the cells were 5-10 fold higher than those of TGF-β1 (Fig. 1a). The intracellular levels of both cytoplasmic isoforms of Hsp90 did not appear to differ between the two lines (Fig. 1f).

### Effect of extracellular TGF-β and Hsp90 on adhesion and migration of SW480 and SW620 cells

**Fig. 2:**
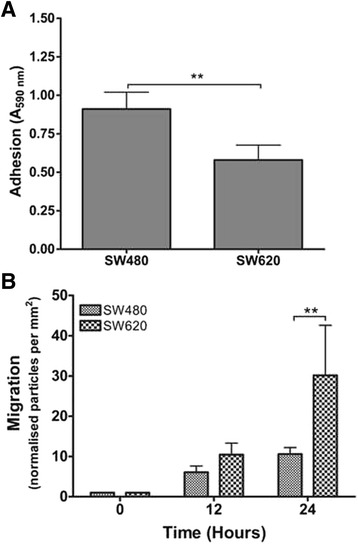
Comparison of adhesion and migration in SW480 and SW620 genetically paired colon cancer cell lines. **a** Adhesion of SW480 and SW620 cells was assessed by absorbance at 590 nm (y-axis) of crystal violet stained adherent cells and a comparison between the adhesion capability of the cells after 8 h was compared. **b** A Comparison of migration capability of SW480 and SW620 cells measuring the cells occupying the wound area after 0, 12 and 24 represented as normalised particles per area over 24 h relative to particles at 0 h (calculated using ImageJ). Graphs are representative of three independent experiments carried out in triplicate showing consistent results. Error bars indicate the standard error in the mean A students *t*-test was performed using GraphPad Prism 4.03 software to assess statistical significance where *n* = 3. (**p < 0.01, ***p < 0.001)

We next examined the effect of the TGF-β pathway and Hsp90 on adhesion and migration in vitro, both of which are biological processes crucial for metastasis. In particular it has been reported that changes in the cytoskeleton and extracellular matrix proteins as well as weak adhesions may cause an increase in migration and may be required for cell motility, revealing that adhesion and migration are two sides of the same coin in terms of metastasis [40–42]. Our analyses revealed that SW480 cells displayed a significantly higher level of adhesion compared to SW620 cells after 8 h incubation (A_590_ values of 0.91 ± 0.27 vs. 0.58 ± 0.24, respectively; p = 0.0027; Fig. 2a), while SW620 cells appeared to be nearly three-fold more migratory than SW480 cells after 24 h (30.15 ± 12.43 vs. 10.58 ± 1.65 particles per mm^2^, respectively; p = 0.0016; Fig. 2b). These opposing trends between the cell lines stand to reason and lend weight to the hypothesis that if there is less adhesion of the cells they are more likely to migrate [40–42]. The migration results also agree with published in vivo studies where SW620 cells were found to be more migratory when injected into mice and analysed for liver metastases [43]. The decreased adhesion and increased migration of SW620 cells observed in this study therefore validates the use of the SW480-SW620 system as an in vitro model of cancer progression.

**Fig. 3:**
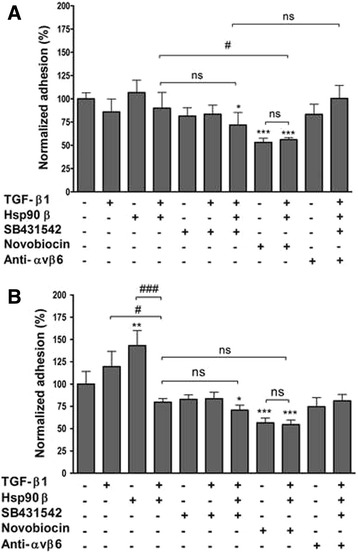
The effect of addition or inhibition of TGF-β and Hsp90 on adhesion in the paired SW480 and SW620 colon cancer cell lines. SW480 **a** and SW620 **b** cells were treated with 2 ng/ml TGF-β1, 20 ng/ml Hsp90β, 100 nM SB431542, 100 μM novobiocin and 10 μg/ml αvβ6 integrin blocking antibody (anti- αvβ6) singly or in combinations as indicated on the x-axis. Absorbance at 590 nm of crystal violet stained adherent cells was normalized to the untreated control, given as 100%, and depicted by the solid horizontal line in the graph, showing the changes in cell adhesion for each treatment. All graphs are representative of the average of three independent experiments performed in triplicate and error bars indicate the standard error in the mean. Statistical analysis was performed using GraphPad Prism 4.03 software. A two-way analysis of variance (ANOVA) with Bonferroni post-test was performed and significance between untreated cells and those after each treatment (indicated by asterisks) and between particular treatments (indicated by hashes) are shown (*p < 0.05, **p < 0.01, ***p < 0.001, ^#^ *p* < 0.05, ^###^ *p* < 0.001, ns – not significant)

The effect of addition or inhibition of TGF-β1 and Hsp90β on the adhesion of SW480 and SW620 cells was analysed by normalisation to untreated cells (taken as a 100%) as depicted in Fig. 3. In the assessment of adhesion, migration and AIG of SW480 and SW620, TGF-β1 and Hsp90β were used at 2 and 20 ng/ml, which corresponded to the levels of proteins detected in spent media (Fig. 1). Treatment with the TGF-βRI inhibitor SB431542 and the C-terminal Hsp90 inhibitor novobiocin was carried out at 100 nM and 100 μM, respectively, in all cases, as these concentrations were not toxic to either cell line (data not shown). Adhesion studies in SW480 cells (Fig. 3a), at 8 h post seeding, revealed that treatment with TGF-β1 did not significantly affect cell adhesion in SW480 cells in comparison to the untreated cells (85.90 ± 13.98% vs. 100.00 ± 8.42% adhesion, respectively). Similarly, Hsp90β treatment did not have any effect on the adhesion of SW480 cells (106.51 ± 13.41% adhesion relative to control). Treatment with a combination of TGF-β1 and Hsp90β, known to bind the common receptor TGF-βRI [31], again showed no significant change in the adhesion of SW480 cells (89.94 ± 16.93%) compared to untreated cells. Inhibiting the receptor TGF-βRI with SB431542, either with or without the addition of exogenous TGF-β1, also had no significant effect on the adhesion of SW480 cells (83.44 ± 9.72% and 81.52 ± 8.77%, respectively). On the other hand, when SB431542 treatment was combined with both TGF-β1 and Hsp90β, this caused a decrease in the adhesion of SW480 (71.80 ± 13.55%) in comparison to the untreated cells, although this was not significantly different to the effect seen after treatment with the two proteins TGF-β1 and Hsp90β alone. The inhibition of Hsp90 by the addition of novobiocin caused a significant decrease in the adhesion of SW480 cells relative to the normalised untreated control (53.09 ± 4.54%). The addition of exogenous Hsp90β and TGF-β1 in combination with novobiocin was not able to overcome the inhibition of total Hsp90 by the drug in terms of adhesion (56.08 ± 2.19%), although it was observed that this decrease in adhesion was significantly less than TGF-β1 and Hsp90β treatments alone. Since αvβ6 integrin is known to be linked to the TGF-β pathway as an alternate TGF-β1 receptor [44], we assessed the effect of inhibition of this integrin on adhesion. We found that treatment with a blocking antibody against αvβ6 (10 μg/ml) had no significant effect on the adhesion of SW480 cells on its own (83.29 ± 10.75%), but that the antibody reversed the decrease in adhesion by TGF-β1, Hsp90β and SB431542 when combined with these treatments (100.29 ± 14.04%), in the SW480 cells.

The effect of addition or inhibition of TGF-β and Hsp90 on adhesion was also assessed in SW620 cells (Fig. 3b), revealing that, in a number of cases, the metastasis-derived cell line did not respond in the same way as the SW480 primary tumour-derived line to these treatments. We found that, unlike in SW480 cells, addition of TGF-β1 caused a subtle increase in the adhesion of SW620 cells compared to the untreated control (119.63 ± 17.16 vs. 100.00 ± 14.30% adhesion). On the other hand, treatment with Hsp90β caused a significant increase in adhesion of SW620 cells over the control (142.99 ± 17.13%, p < 0.01) which was once more not seen for SW480 cells. Unexpectedly, despite the fact that they each increased adhesion, treatment with a combination of TGF-β1 and Hsp90β caused a significant decrease in the adhesion of SW620 cells (79.69 ± 4.09%). This decrease in adhesion of SW620 cells was even greater when comparing Hsp90β treatment alone to the combination treatment with TGF-β1 (*p* < 0.001). As was the case for SW480 cells, inhibition of TGF-βRI with SB431542, either with or without the addition of exogenous TGF-β1, did not have a significant effect on the adhesion of SW620 cells in comparison to the untreated cells (83.55 ± 7.43% and 82.91 ± 5.01%, respectively). Again, it was only when SB431542 treatment was combined with both TGF-β1 and Hsp90β, that a significant decrease in the adhesion of the cells was observed (70.68 ± 5.56%). Similar to the effect seen for SW480 cells, this effect was not significantly different to that seen after treatment with the two proteins TGF-β1 and Hsp90β alone. As was the case for SW480 cells, the inhibition of Hsp90 by novobiocin caused a marked decrease in the adhesion of SW620 cells relative to the normalised untreated control (56.45 ± 5.35%, p < 0.001). The addition of exogenous Hsp90β and TGF-β1 in combination with novobiocin was once more not able to overcome the inhibition of Hsp90 on adhesion (54.53 ± 5.02%). As for SW480 cells, treatment with a αvβ6 blocking antibody had no significant effect on the adhesion of SW620 cells on its own (74.68 ± 10.12%); however, when combined with TGF-β1, Hsp90β and SB431542, the antibody reversed the decreased adhesion by a combination of the latter three treatments alone (81.25 ± 7.02%).

**Fig. 4:**
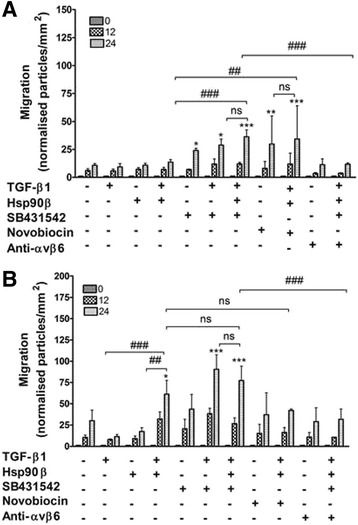
The effect of addition or inhibition of TGF-β and Hsp90 on migration of the paired SW480 and SW620 colon cancer cell lines. SW480 **a** and SW620 **b** cells were treated with 2 ng/ml TGF-β1, 20 ng/ml Hsp90β, 100 nM SB431542, 100 μM novobiocin and 10 μg/ml αvβ6 integrin blocking antibody (anti- αvβ6) singly or in combinations as indicated on the x-axis and the effect of such treatment on migration assessed. All graphs are representative of the average of three independent experiments performed in triplicate and error bars indicate the standard error in the mean. Statistical analysis was performed using GraphPad Prism 4.03 software. A two-way analysis of variance (ANOVA) with Bonferroni post-test was performed and significance between untreated cells and those after each treatment (indicated by asterisks) and between particular treatments (indicated by hashes) are shown (*p < 0.05, **p < 0.01, ***p < 0.001, ^##^ *p* < 0.01, ^###^ *p* < 0.001, ns – not significant)

Migration analysis was performed and the wound area covered by cells calculated using ImageJ [particles per area (mm^2^)] and normalised to each treatment at 0 h (given as 1) in each of the two cell lines (Fig. 4a and b). In this case, statistical analysis was carried out between treatments at 24 h as this was when the greatest migration was observed. For the SW480 cells (Fig. 4a), treatment with TGF-β1 alone, Hsp90β alone as well as the combination of TGF-β1 with Hsp90β, caused no significant changes in the migration of the cells relative to the untreated control (9.26 ± 3.08, 10.72 ± 1.71 and 13.44 ± 2.28, respectively vs. 10.58 ± 1.65 normalised particles per mm^2^/NPPM). In contrast to this, the inhibition of the TGF-βRI using SB431542 caused a two-fold increase in migration of SW480 cells in comparison to the untreated cells (23.93 ± 2.07 NPPM). The effect of addition of TGF-β1 in combination with SB431542 on migration (28.67 ± 5.58 NPPM) did not differ significantly to that of treatment with SB431542 alone, suggesting that the addition of TGF-β1 was unable to overcome the inhibition of the canonical TGF-β/TGF-βR pathway in these cells. The addition of both TGF-β1 and Hsp90 with SB431542 treatment, on the other hand, caused a significant three-fold increase compared the untreated cells (36.18 ± 6.24 NPPM). This treatment was also greatly increased in comparison to the combination TGF-β1 and Hsp90β treatment (*p* < 0.001). The effect of the combination of TGF-β1, Hsp90 and SB431542 on migration, however, was not significantly different to that of SB431542 treatment alone. Novobiocin treatment caused an increase in migration in SW480 cells relative to the untreated control (29.76 ± 25.17 NPPM). The effect on migration of TGF-β1 and Hsp90β in combination with the novobiocin (34.25 ± 29.79 NPPM) did not differ significantly in comparison to novobiocin treatment alone, suggesting that the addition of endogenous Hsp90β and TGF-β1 could not overcome the inhibition of Hsp90 by novobiocin. These treatments did however cause a substantial increase in migration, in comparison to the treatment using a combination of only TGF-β1 and Hsp90β (p < 0.01). This highlights that it is the novobiocin specifically that is causing the increase in migration. The inhibition of the αvβ6 integrin with a blocking antibody (10 μg/ml) alone did not have any effect on the migration of SW480 cells (11.33 ± 5.20 NPPM), including when TGF-β1, Hsp90β and SB431542 were added (11.67 ± 1.25 NPPM), effectively returning migration to baseline compared to treatment with TGF-β1, Hsp90β and SB431542 alone. This suggests that αvβ6 integrin may be an alternate receptor mediating migration in response to a combination of TGF-β1 and Hsp90β in these cells.

When analysing the migration of SW620 cells (Fig. 4b), it was found that treatment with either TGF-β1 or Hsp90β caused minor decreases in the migration of these cells in comparison to the untreated cells (11.46 ± 2.53 and 17.30 ± 4.66 vs. 30.15 ± 12.43 NPPM, respectively). On the other hand, treatment with a combination of TGF-β1 and Hsp90β caused a statistically significant two-fold increase in migration of SW620 cells in comparison to untreated cells (61.21 ± 16.24 NPPM). This increase in migration of SW620 cells caused by the combination treatment was significantly higher than either TGF-β1 or Hsp90β treatment alone (p < 0.001 and p < 0.01, respectively). Unlike in SW480 cells, SB431542 treatment alone did not cause a significant change in the migration of SW620 cells relative to the untreated control (43.73 ± 17.25 NPPM). However, similarly to SW480 cells, treatment of SW620 cells with a combination of SB431542 and TGF-β1 as well as a combination of SB431542, TGF-β1 and Hsp90β caused a substantial increase in migration (90.50 ± 17.03 and 77.28 ± 16.89 NPPM, respectively). This increase in migration was however not significantly different to the combination treatment of TGF-β1 and Hsp90β alone. These data suggest that the pro-migratory effect of the combination of TGF-β1 and Hsp90β is not disrupted by inhibition of the receptor, suggesting that the canonical pathway is not being used. Treatment with novobiocin alone and in combination with TGF-β1 and Hsp90β had no significant effect on the migration of SW620 cells relative to the untreated control (37.27 ± 25.75 and 42.15 ± 1.82 NPPM, respectively), unlike in SW480 where the drug caused a substantial increase in migration. While disruption of αvβ6 integrin using a blocking antibody did not cause any change in the migration of SW620 cells (28.98 ± 16.34 NPPM), this was able to overcome the stimulation of migration caused by a combination of TGF-β1, Hsp90β and SB431542, (31.71 ± 12.16 NPPM for treatment with αvβ6 integrin + TGF-β1 + Hsp90β + SB- 431542), thereby returning migration to a level comparable with untreated cells. This trend was the same as that observed in SW480 cells.

### Effect of extracellular TGF-β and Hsp90 on anchorage-independent growth (AIG) of SW480 and SW620 cells

**Fig. 5:**
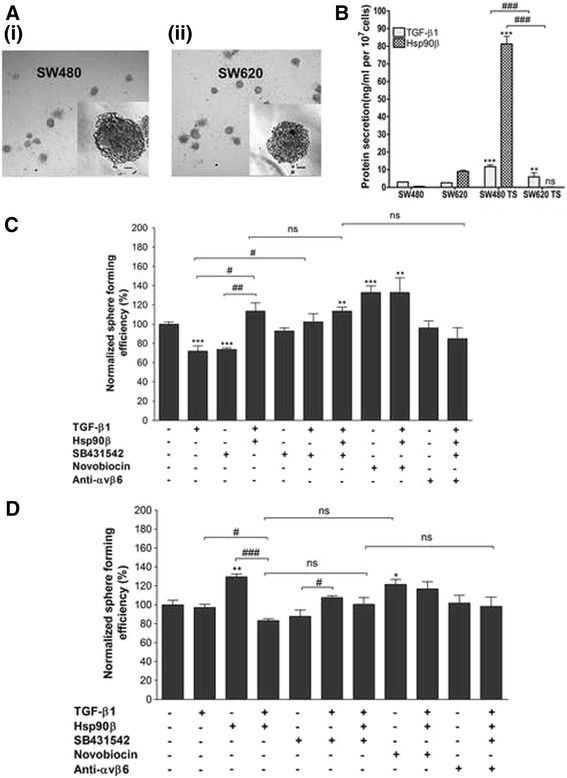
Investigation of the role of TGF-β and Hsp90 in anchorage-independent growth of SW480 and SW620 cells. **a** Photograph of tumourspheres formed by SW480 (i) and SW620 (ii) cells taken under a light microscope at 100x magnification. Scale bars indicate 100 μm. **b** Comparison of TGF-β1 and Hsp90β secretion by SW480 primary and SW620 secondary tumour-derived cells grown adherently and in suspension using a DuoSet ELISA kit (R and D systems) and sandwich ELISA method, respectively. Data shown are representative of three individual experiments carried out in triplicate and showing consistent results. Statistical significance was assessed using GraphPad Prism 4.03 software by means of a two-way analysis of variance (ANOVA) with Bonferroni post-test where *n* = 3. Comparisons in terms of the levels of proteins between adherent cells and tumoursphere (within cell lines) is indicated by asterisks (*), while comparisons between cell lines is indicated by hashes (#). **c** and **d** Analysis of the effect of addition or inhibition of TGF-β or Hsp90 on tumoursphere formation. Sphere forming efficiency (percentage of the total number of cells seeded that are able to form tumourspheres after 7 days) of SW480 **c** and SW620 cells **d** was normalized to that of untreated cells for each cell line (taken as 100%). Error bars indicate the standard error in the mean where *n* = 4. Statistical significance was assessed using GraphPad Prism 4.03 software by means of a two-way analysis of variance (ANOVA) with Bonferroni post-test and significance between untreated cells and those after each treatment (indicated by asterisks) as well as between particular treatments (indicated by hashes) are shown (*p < 0.05, **p < 0.01, ^#^ *p* < 0.05, ^##^ *p* < 0.01, ^###^ *p* < 0.001)

Since the ability of malignant cells to survive and grow anchorage-independently is a requirement for metastasis, we investigated the secretion of and response to TGF-β1 and Hsp90β of the paired colon cancer cell lines under such conditions. The cell lines were grown in suspension as well defined three-dimensional structures known as tumourspheres, with both cell lines forming spheres of similar morphologies and size (Fig. 5a). When comparing the secretion of TGF-β1 and Hsp90β by these tumourspheres (Fig. 5b) we found that SW480 tumourspheres produced much higher levels (*p* < 0.001) of both proteins in the spent media compared to SW620 tumourspheres (TGF-β1: 11.68 ± 1.03 vs. 5.85 ± 2.35 ng/ml, respectively; Hsp90β: 81.28 ± 0.43 vs. 0.10 ± 0.23, respectively) as well as to either cell line grown adherently (TGF-β1: 2.97 ± 0.05 ng/ml in SW480, 2.52 ± 0.04 ng/ml in SW620; Hsp90β: 0.44 ± 0.18 ng/ml in SW480, 8.96 ± 0.66, in SW620 cells).

We next assessed the effect of addition or inhibition of TGF-β1 and Hsp90β on the ability of both cell lines to form tumourspheres as a measure of anchorage-independent growth (Fig. 5c). Sphere forming efficiency (SFE) was defined as the percentage of the total number of cells seeded that are able to form tumourspheres after 7 days and this was normalized to that of untreated cells for each cell line (taken as 100%). In SW480 cells (Fig. 5c), we found that addition of either TGF-β1 or Hsp90β alone caused a statistically significant decrease in SFE compared to untreated cells (72.00 ± 9.60 and 74.13 ± 2.44%, respectively vs. 100.00 ± 6.40%; p < 0.001 in each case). On the other hand, a combination of Hsp90β and TGF-β1 led to a significant increase in SFE (108.27 ± 6.65%) relative to SW480 cells treated with either protein alone (p < 0.05 for TGF-β1, p < 0.01 for Hsp90β), returning SFE to the level observed in untreated cells. While the addition of SB431542 alone had no significant effect on SFE in these cells (94.93 ± 1.85%), the inhibitor was able to reverse the effect of TGF-β1 on SFE (107.73 ± 6.66%). Interestingly, the combination of TGF-β1, Hsp90β and SB431542 caused a marked increase in SFE relative to the untreated control (111.47 ± 5.11%, p < 0.01). An even greater increase in SFE was observed for cells treated with the novobiocin (137.07 ± 6.06%, p < 0.001), and this was not affected by the addition of both TGF-β1 and Hsp90β (138.67 ± 22.19%). Since αVβ6 integrin is an alternate receptor in the TGF-β machinery, we assessed the effect of a blocking antibody against this integrin on SFE. While the antibody alone had no effect on SFE of SW480 cells (99.20 ± 9.99%), it was able to reverse the increase in SFE caused by the combination of TGF-β1, Hsp90β and SB431542 (82.66 ± 19.29% for cell treated with the αVβ6 integrin antibody, TGF-β1, Hsp90β and SB431542).

In terms of anchorage-independent growth in the SW620 cell line (Fig. 5d), it was clear that these cells responded very differently not only to addition but also inhibition of TGF-β and Hsp90 compared to the SW480 line and that, in general, these treatments had less of an impact on SFE in SW620 cells. In particular we found that, unlike in SW480 cells, anchorage-independent growth in these cells was not affected by the addition of TGF-β1 (SFE of 97.11 ± 6.00% in treated cells vs. 100.00 ± 11.69% in untreated cells). Hsp90, on the other hand, caused a significant increase in SFE in SW620 cells (129.87 ± 5.21%), a trend which is opposite to that observed in SW480 cells. Interestingly, this increase in SFE upon Hsp90 treatment was reversed when combined with TGF-β1 (84.39 ± 2.00% SFE). Treatment with SB431542 had no real effect on tumoursphere formation either alone (90.56 ± 8.98% SFE) or when combined with TGF-β1 alone (108.67 ± 2.00%) or TGF-β1 with Hsp90β (105.20 ± 4.00%). Unexpectedly, inhibition with novobiocin also increased AIG in SW620 cells (120.23 ± 8.73%) in the same manner as Hsp90β addition but this effect was no longer significant when the drug was combined with TGF-β1 and Hsp90β (119.85 ± 10.74%). It is important to note however that this inhibitor is not specific to the Hsp90β isoform or extracellular Hsp90. An αVβ6 integrin blocking antibody had no effect on SFE of SW620 cells either alone (96.34 ± 5.82%), or when combined with TGF-β1, Hsp90β and SB 431542 (91.71 ± 5.95%).

### Effect of Hsp90 and TGF-β1 pre-treatment under AIG conditions on chemosensitivity to colon cancer chemotherapeutics

**Fig. 6:**
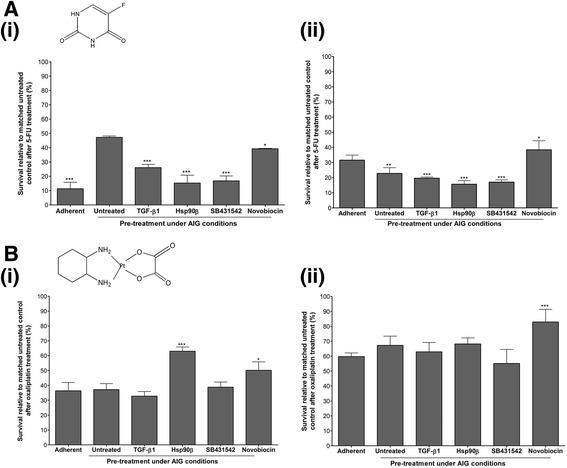
Effect of pre-treatment of paired colon cancer cell lines by the addition or inhibition of TGF-β or Hsp90 under anchorage independent conditions on subsequent sensitivity to colon chemotherapeutics. SW480 (i) and SW620 (ii) cells were treated with either 2 ng/ml TGF-β1, 20 ng/ml Hsp90β, 100 nM SB431542 or 100 μM novobiocin upon seeding in a tumoursphere assay. After 7 days, tumourspheres were reseeded into regular adherent growth conditions and treated with 75 μM 5-fluorouracil **a** or 550 μM oxaliplatin **b** for 72 h. Cell viability was assessed compared to an untreated control for each pre-treatment using a MTT Cell Proliferation kit. Data are representative of two individual experiments carried out in triplicate and showing consistent results. Statistical significance was assessed by means of a two-way analysis of variance (ANOVA) with Bonferroni post-tests relative to the non-pre-treated control using GraphPad prism where *n* = 3 (*p < 0.05, **p < 0.01)

We assessed the effect of pre-treatment under AIG conditions by either addition or inhibition of TGF-β1 and Hsp90 on chemosensitivity of SW480 and SW620 cells to oxaliplatin and 5-FU (Fig. 6). Pre-treatment was carried out under AIG conditions since this has been demonstrated to enrich for cancer stem cells (CSC) by ourselves and others [38, 45] and this sub-population is reportedly resistant to traditional chemotherapeutic agents due to the overexpression of several drug transporters [46]. The chemosensitivity assay used high concentrations of the colon cancer drugs as previously described [47], namely 550 μM 5-FU and 75 μM oxaliplatin, in order to see any chemoresistance which may result from the various pre-treatments. In the assay, pre-treatment with TGF-β1, Hsp90, SB 431542 or novobiocin was carried out upon seeding into AIG conditions. The resultant tumourspheres after 7 days of culture were dissociated and seeded into regular culture conditions, allowed to adhere overnight, and then treated with vehicle control (representing “untreated” cells), 5-FU or oxaliplatin. For each pre-treatment, viability was calculated relative to its own vehicle-treated control (taken as 100%). A comparison was also made with untreated cells (i.e. not pre-treated) that had previously either been grown adherently (first bar in each graph of Fig. 6) or been cultured as tumourspheres and then dissociated (second bar in each graph of Fig. 6). In each case, statistical significance was assessed in comparison to cells with had been cultured as tumourspheres but not pre-treated (second bar in each graph). We report that pre-culturing as tumourspheres under AIG conditions appears to infer chemoresistance of SW480 primary tumour cells to 5-FU (47.16 ± 0.54% survival for tumoursphere-derived untreated cells vs. 11.27 ± 2.64% for adherently cultured untreated cells, Fig. 6ai). This was expected since a previous study by [47] reported that colon CSC are 60-fold more resistant to 5-FU. In terms of pre-treatment during AIG, we found that TGF-β1, Hsp90β, SB431542 and novobiocin all significantly increased chemosensitivity of SW480 cells to 5-FU (26.04 ± 1.30%, 15.31 ± 3.15%, 16.79 ± 1.96% and 39.20 ± 0.17% survival, respectively, Fig. 6ai).

Converse to that observed for SW480 cells, pre-culture as tumourspheres rendered SW620 cells significantly more sensitive to 5-FU (22.81 ± 2.17 for tumoursphere-derived cells vs. 31.53 ± 1.94% for adherent cells, Fig. 6aii). On the other hand, similar trends were noted in the SW620 cells compared to the SW480 cells in terms of the effect of pre-treatment with TGF-β1, Hsp90β and SB431542, though the resultant chemosensitivity was less pronounced (19.63 ± 0.45%, 15.69 ± 1.38% and 17.04 ± 0.85%, respectively, Fig. 6aii). The major difference between the cell lines in terms of pre-treatment was the effect of novobiocin which, unlike in SW480 cells, significantly decreased the sensitivity of SW620 cells to 5-FU (38.36 ± 3.48% survival).

We report that, unlike for 5-FU, AIG pre-culture did not significantly influence the chemosensitivity of SW480 cells to oxaliplatin (37.16 ± 2.30% survival in tumoursphere-derived cells vs. 36.35 ± 3.24% in adherent cells, Fig. 6bi). This was unexpected since [47] reported that colon CSC are 15-fold more resistant to oxaliplatin than bulk colon tumour cells. The various pre-treatments had less of an effect on chemosensitivity in general of SW480 cells to oxaliplatin compared to 5-FU. In particular, pre-treatment with TGF-β1 and SB431542 did not influence chemosensitivity to oxaliplatin (37.72 ± 1.82%, 38.74 ± 2.02% survival, respectively) while Hsp90β and novobiocin pre-treatment rendered SW480 cells significantly more resistant to the drug (63.00 ± 1.63% and 82.91 ± 4.90% survival, respectively, Fig. 6bi), the latter effect being opposite to that observed for 5-FU.

In the case of SW620 cells, as for SW480 cells AIG pre-culture did not influence the chemosensitivity of the cells to oxaliplatin (67.22 ± 3.56% in tumoursphere-derived cells vs. 59.73 ± 2.42% in adherent cells, Fig. 6bii). In terms of pre-treatment, only SB431542 treatment had a significant effect on sensitivity to oxaliplatin, causing a slight increase in sensitivity of SW620 cells (55.09 ± 5.03% survival), with no significant effects on chemosensitivity noted for pre-treatment with TGF-β1, Hsp90β or novobiocin (62.93 ± 3.62%, 68.17 ± 2.35% and 82.91 ± 4.90% survival, respectively, Fig. 6bii).

## Discussion

The role of TGF-β in cancer progression is notoriously duplicitous, with the chemokine acting as a tumour suppressor in the early stages of carcinogenesis but a promoter of metastasis in the later stages [14]. The SW480/SW620 cell lines represent an in vitro model for the changes associated with the acquisition of a metastatic phenotype since the cell lines are derived from a primary adenocarcinoma of the colon (SW480) and its lymph node metastasis (SW620) from the same patient [48]. It was therefore interesting to observe the levels of the various components of the TGF-β pathway in these cells. The majority of studies regarding the role of the TGF-β in colon cancer reported in the literature have been carried out in resected tumours and their respective metastases and ours is thus the first study to our knowledge comparing the TGF-β1 machinery in paired colon cancer cell lines. We report that there are key differences in the TGF-β1 pathway between the SW480 and SW620 cell lines. In particular, the SW480 primary tumour-derived line displays a higher level of the receptor TGF-βRII, while the SW620 secondary tumour-derived line displays higher intracellular TGF-βI. These findings agree with published reports in that a low level of TGF-βR (both TGF-βRI and TGF- βRII) has been demonstrated to correlate with disease progression [49] and high levels of TGF-β1 in colon cancer have been correlated with a poor disease prognosis in a clinical setting, specifically increasing metastasis and general invasiveness [50, 51]. Furthermore, the increase in nuclear pSmad2/3 observed in SW620 cells in this study suggested that the pathway may be constitutively active in the metastasis-derived line.

Since TGF-β1 and Hsp90β are able to bind the same receptors [31], we explored in detail the consequences of a synergy between these processes on adhesion, migration and AIG. We report that exogenous TGF-β1 has no effect on the level of adhesion or migration in either cell line but that the addition of Hsp90β increases the adhesion of SW620 secondary tumour but not SW480 primary tumour-derived cells, without affecting migration. The finding that TGF-β1 has no effect on migration was unexpected since a number of studies have suggested that the protein increases migration, in particular in lung cancer [52], hepatocellular carcinoma [32] and breast cancer cell lines [33] using a range of techniques. Similarly, extracellular Hsp90 has been shown to stimulate migration in glioblastoma (A-172) and fibrosarcoma (HT1080) cell lines [53], which was not the case in our study. Interestingly, we found that the effect of addition of TGF-β1 and Hsp90β on AIG is opposite in the two cell lines in that the exogenous addition of each of these proteins inhibits the formation of tumourspheres in SW480 primary tumour-derived cells, but has either no effect (in the case of TGF-β1) or stimulates tumoursphere formation (in the case of Hsp90β) in the SW620 metastasis-derived line. Overall, the latter findings suggest that cells in a primary tumour may respond differently to cytokine signalling by TGF-β1 and Hsp90β compared to cells in a secondary tumour in terms of metastatic behaviour, in particular adhesion, migration and AIG.

In contrast to the effect of the proteins alone, we found that treatment with a combination of TGF-β1 and Hsp90β caused a significant decrease in the adhesion and corresponding increase in migration of SW620 cells while having no effect on SW480 cells. In addition, we noted that the effect of TGF-β1 and Hsp90β alone on AIG is opposite to that when combined in the SW480 cell line. These findings led us to suspect a synergy between TGF-β1 and Hsp90β in mediating adhesion, migration and AIG, which has not previously been suggested in the literature.

We note that, in SW480 cells, the greatest effect on adhesion in the cell lines was caused by treatment with the Hsp90 inhibitor novobiocin, which both alone and in combination with TGF-β1 and Hsp90β, decreased the level of adhesion in both cell lines, suggesting that it is Hsp90β and not TGF-β1 which plays the more important role in regulating adhesion in these cancer cells. Conversely, in terms of migration, it appears that it is TGF-βRI which is the major regulatory protein, particularly in SW480 cells as SB431542 treatment alone, in combination with TGF-β1 or with TGF-β1 and Hsp90β increases migration in SW480 cells. On the other hand, in SW620 cells this effect is not seen with the drug alone but only when it is combined with TGF-β1 or with TGF-β1 and Hsp90β, suggesting that these cells do not require inhibition of the canonical TGF-β pathway in order to stimulate migration in the presence of a combination of TGF-β1 and Hsp90β as evidenced by the fact that these two proteins alone are able to achieve this. This would allow them to respond more effectively to the pro-metastasis stimulus of a combination of Hsp90β and TGF-β1 and this could account for the increased migratory capacity of these cells. Contrary to the findings in our study, previous reports have demonstrated that SB431542 inhibits lung (A549) and glioma (D54MG) cancer cell migration [52, 54], in particular that the compound is able to reverse the increased migration associated with TGF-β1 treatment of A549 cells [52]. It is unclear whether these differences are due to the different migration assays used, in that our study assessed linear migration via a wound healing assay whereas Halder et al and Hjelmeland et al [52, 54] used a transwell assay, or whether the differences reflect the different roles of the TGF-βRI-mediated pathway in cancer cell lines of different origins.

The most intriguing finding in this study was that a combination of TGF-β1, Hsp90β and the TGF-βRI inhibitor SB431542 markedly decreased the level of adhesion, while increasing migration and AIG of both SW480 and SW620 cells, revealing a clear trend towards metastatic behaviour of the cells in response to this combination of treatments. These findings suggested that an alternate receptor (in place of TGF-βRI) was acting in the presence of TGF-β1 when the canonical TGF-β1 pathway was inhibited by treatment with SB431542. We considered αvβ6 integrin as a potential candidate for this alternate receptor as signalling by TGF-β1 via αvβ6 integrin has been demonstrated in a range of carcinoma cell lines [44]. Indeed, we found that treatment with a blocking antibody against αvβ6 integrin had no effect on adhesion, migration or AIG of either cell line on its own, but that the antibody reversed the decrease in adhesion and increase in migration and anchorage-independent growth upon combination treatment with TGF-β1, Hsp90β and SB431542. Taken together, this suggests a role for αvβ6 integrin in mediating this unique effect of the two proteins on these cellular processes.

Finally, we examined a potential role for TGF-β1 and/or Hsp90β in conferring chemoresistance of the paired SW480 and SW620 colon cell lines to the chemotherapeutics 5-FU and oxaliplatin. Pre-treatment was carried out under AIG conditions since this assay is known to enrich for CSC [45], a sub-population that has been suggested to be resistant to traditional chemotherapeutics [46]. Indeed, culturing of SW480 cells under AIG conditions rendered them more resistant to 5-FU, although this trend was not observed for either SW620 cells in terms of sensitivity to 5-FU or for either SW480 or SW620 cells in terms of sensitivity to oxaliplatin. In addition, we report that, although the effect of TGF-β1 and Hsp90β on tumoursphere forming ability differs greatly between the SW480 and SW620 cells, the effect of pre-treatment of these cells under AIG conditions by the addition or inhibition of these proteins on chemosensitivity to 5-FU and oxaliplatin were relatively similar overall. The major exception to this was in the effect of novobiocin pre-treatment, which differed greatly between the cell lines and the two drugs tested. Importantly, from a therapeutic viewpoint, none of the pre-treatments increased chemosensitivity of the colon cancer cell lines to either 5-FU or oxaliplatin to below the level of untreated cells cultured adherently.

## Conclusions

In summary, we report that there are key differences in the TGF-β1 pathway between the SW480 cell line derived from a primary colon adenocarcinoma and its SW620 lymph node metastasis-derived counterpart, in particular that the pathway may be constitutively active in the metastasis.

**Fig. 7:**
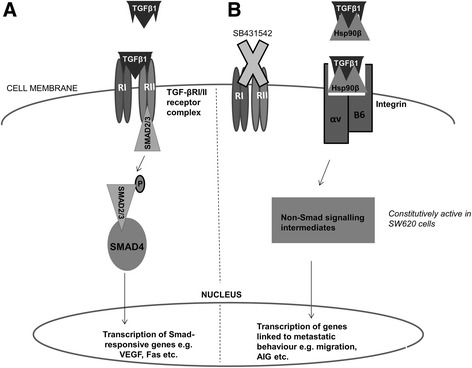
Model describing the effect of a synergy between extracellular TGF-β1 and Hsp90β on downstream signalling in colon cancer cells. **a** In the presence of TGF-β1 alone, binding to the heterotetrameric TGF-βRI/II receptor complex triggers phosphorylation of Smad2/3 and subsequent activation of Smad4, whereupon the complex moves into the nucleus and triggers transcription of canonical Smad-responsive genes such as VEGF (tumour promoting) and Fas (tumour suppressing). **b** In the presence of both TGF-β1 and Hsp90β in the extracellular space, when binding of TGF-β1 to the TGF-βRI/II receptor complex is inhibited using SB431542, TGF-β1 and Hsp90β instead bind to αvβ6 integrin, triggering as yet unknown downstream non-Smad signalling pathways, culminating in the transcription of genes which promote metastatic behaviours including migration and anchorage-independent growth (AIG). This alternate pathway does not require inhibition of TGF-βRI/II and is constitutively active in SW620 cells, which represent a later stage of colon cancer compared to SW480 cells

We suggest that an alternate pathway to the canonical TGF-βR1/II-Smad2/3 (Fig. 7a) may be operating in the cells when TGF-β1 occurs together with Hsp90β (Fig. 7b) and that this pathway promotes metastatic behaviour in terms of adhesion, migration and AIG when the canonical TGF-β pathway is inhibited, particularly in SW480 cells, which are a model for early stage colon cancer. In all cases, αvβ6 integrin was identified as the potential alternate receptor for TGF-β1 mediating these metastatic processes in the presence of Hsp90β. Interestingly, in the case of migration, it would appear that the alternate TGF-β1 pathway (Fig. 7b) is already active in SW620 cells without requiring inhibition by SB431542 in these cells which represent a later stage of the disease. If this is the case, then it is feasible that the activation of such a pathway which allows for an increased ability to respond to the pro-metastasis signalling of a combination of TGF-β1 and Hsp90β could be responsible for the spread of cancer from the primary tumour to the lymph node in the patient from whom the genetically paired cell lines were derived. This study therefore suggests a potential biological relevance for the previously proposed role of Hsp90 in regulating TGF-β signalling.

## Additional file

Additional file 1: Western blot analysis of the intracellular levels of TGF-β1 in SW480 and SW620 cells compared to a histone loading control. Full-length versions of the western blots shown in Fig. 1c. A) SW480 and SW620 whole cell lysates were probed for TGF-β1 using mouse anti-human TGF-β1 (sc-65378, Santa Cruz). Purified recombinant TGF-β1 (Biolegend) in its acid-activated form was included as a positive control (+). B) The membrane was reprobed for histone as a loading control using rabbit anti-human histone (9715 L, Cell Signalling Technologies). (TIF 1216 kb)

## Abbreviations

5-FU: 5-fluorouracil
AIG: Anchorage-independent growth
BMPs: Bone morphogenetic proteins
CSCs: Cancer stem cells
GDFs: Growth and differentiation factors
Hsp90: Heat shock protein 90
NPMM: Normalized particles per mm^2^
SFE: Sphere forming efficiency
TGF-β: Transforming growth factor-beta
TGF-βRI/II: Transforming growth factor-beta receptor I/II
