# Supplementary figures and images for: Extracellular Hsp90 and TGFβ regulate adhesion, migration and anchorage independent growth in a paired colon cancer cell line model
Source: BMC Cancer. 2017 Mar 16;17:202. doi: 10.1186/s12885-017-3190-z (PMC5356307; doi:10.1186/s12885-017-3190-z)

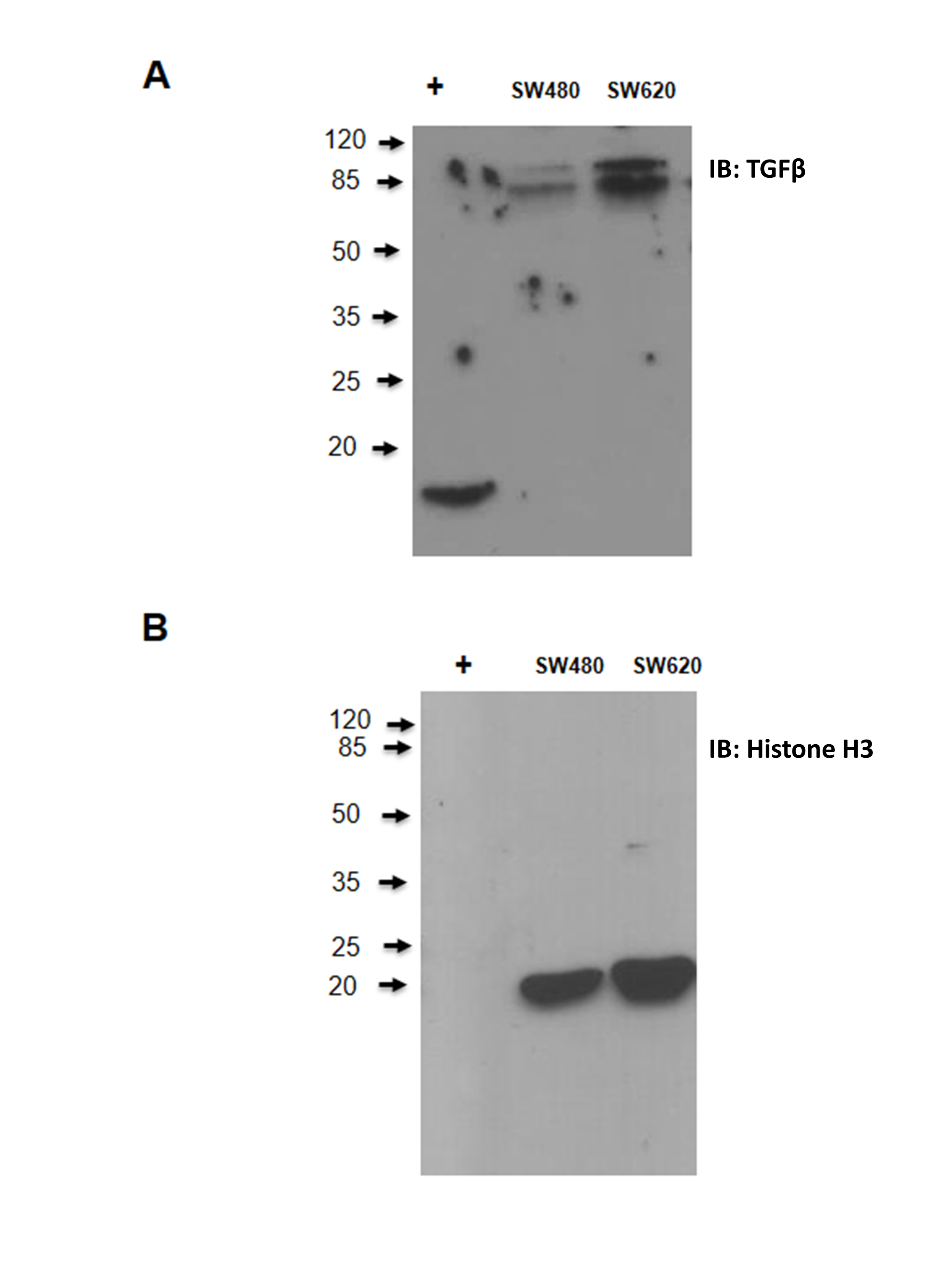

Supplement: Additional file 1: — Western blot analysis of the intracellular levels of TGF-β1 in SW480 and SW620 cells compared to a histone loading control. Full-length versions of the western blots shown in Fig. 1c. A) SW480 and SW620 whole cell lysates were probed for TGF-β1 using mouse anti-human TGF-β1 (sc-65378, Santa Cruz). Purified recombinant TGF-β1 (Biolegend) in its acid-activated form was included as a positive control (+). B) The membrane was reprobed for histone as a loading control using rabbit anti-human histone (9715 L, Cell Signalling Technologies). (TIF 1216 kb) [file 12885_2017_3190_MOESM1_ESM.tif]
